# Gene expression profile during seed development of *Bixa orellana* accessions varying in bixin pigment

**DOI:** 10.3389/fpls.2023.1066509

**Published:** 2023-02-15

**Authors:** Yair Cárdenas-Conejo, José Alberto Narváez-Zapata, Víctor Manuel Carballo-Uicab, Margarita Aguilar-Espinosa, Rosa Us-Camas, Pedro Escobar-Turriza, Luca Comai, Renata Rivera-Madrid

**Affiliations:** ^1^ Laboratorio de Agrobiotecnología, Consejo Nacional de Ciencia y Tecnología (CONACYT)-Universidad de Colima, Colima, Mexico; ^2^ Instituto Politécnico Nacional, Centro de Biotecnología Genómica, Reynosa, Tamaulipas, Mexico; ^3^ Unidad de Bioquímica y Biología Molecular de Plantas, Centro de Investigación Científica de Yucatán A.C., Mérida, Yucatán, Mexico; ^4^ Departamento de Estudios de Posgrado e Investigación, Instituto Tecnológico Superior de Calkiní, en el Estado de Campeche, Calkiní, Campeche, Mexico; ^5^ Segunda División de Biotecnología Industrial, Centro de Investigación Científica y Asistencia en Tecnología y Diseño del Estado de Jalisco, Zapopan, Jalisco, Mexico; ^6^ Plant Biology and Genome Center, University of California, Davis, Davis, CA, United States

**Keywords:** *Bixa orellana*, bixin, apocarotenoids, terpenes, pigment gland cells, achiote, secondary metabolism

## Abstract

Diverse morphological, cellular and physiological changes occur during seed maturation in *Bixa orellana* when the seed tissues form specialized cell glands that produce reddish latex with high bixin amounts. Transcriptomic profiling during seed development in three *B. orellana* accessions (P12, N4 and N5) with contrasting morphologic characteristics showed enrichment in pathways of triterpenes, sesquiterpenes, and cuticular wax biosynthesis. WGCNA allows groups of all identified genes in six modules the module turquoise, the largest and highly correlated with the bixin content. The high number of genes in this module suggests a diversification of regulatory mechanisms for bixin accumulation with the genes belonging to isoprene, triterpenes and carotene pathways, being more highly correlated with the bixin content. Analysis of key genes of the mevalonate (MVA) and the 2C-methyl-D-erythritol-4-phosphate (MEP) pathways revealed specific activities of orthologs of *BoHMGR*, *BoFFP*, *BoDXS*, and *BoHDR.* This suggests that isoprenoid production is necessary for compounds included in the reddish latex of developing seeds. The carotenoid-related genes *BoPSY2*, *BoPDS1* and *BoZDS* displayed a high correlation with bixin production, consistent with the requirement for carotene precursors for apocarotenoid biosynthesis. The *BoCCD* gene member (*BoCCD4-4*) and some *BoALDH* (*ALDH2B7.2* and *ALDH3I1*) and *BoMET* (*BoSABATH1* and *BoSABATH8*) gene members were highly correlated to bixin in the final seed development stage. This suggested a contributing role for several genes in apocarotenoid production. The results revealed high genetic complexity in the biosynthesis of reddish latex and bixin in specialized seed cell glands in different accessions of *B. orellana* suggesting gene expression coordination between both metabolite biosynthesis processes.

## 1 Introduction

*Bixa orellana*, also known as achiote or lipstick tree, is a crop of great agroindustrial interest because it produces high quantities of the commercial dye known as annatto (E160b), which is composed mainly of the C_25_ apocarotenoid bixin (Rivera-Madrid et al., 2006). Several *B. orellana* accessions have been phenotypically characterized by searching for traits to improve the pigment yields in their seeds (Valdez-Ojeda et al., 2010; Rodríguez-Ávila et al., 2011a; Trujillo-Hdz et al., 2016; Tamayo-García et al., 2022). Sixteen achiote accessions were grouped according to their flower color, fruit color and bixin production into three main groups (groups A, B and C) (Trujillo-Hdz et al., 2016). A *de novo* transcriptomic analysis on mature seeds of the higher bixin producer accession (“Peruana Roja”, group C) revealed a wide diversity of carotene and carotenoid genes, including several carotenoid cleavage dioxygenases (*BoCCDs*), aldehyde dehydrogenases (*BoALDHs*) and methyltransferases (*BoMETs*) (Cárdenas-Conejo et al., 2015).

Bixin biosynthesis genes were identified in the early 2000s, based on a subtractive library constructed from immature seeds, where the apocarotenoid bixin is mainly produced. It was suggested that lycopene is converted to bixin in sequential steps by three types of enzymes, carotenoid dioxygenase (*CCDs*), aldehyde dehydrogenase (*ALDHs*) and methyltransferases (*METs*) (Jako et al., 2002). By using a heterologous system, different *BoCCDs* gene members (*BoCCD1-1*, *BoCCD1-3*, *BoCCD1-4*, *BoCCD4-1*, *BoCCD4-2*, *BoCCD4-3*, and *BoCCD4-4*) can produce bixin aldehyde, a key precursor in bixin production (Cárdenas-Conejo et al., 2015; Carballo-Uicab et al., 2019; Us-Camas et al., 2022). Other genes related to apocarotenoids have also been studied, such as the MET members of the SABATH family (Wei et al., 2021). Specific *SABATH* genes have been correlated with bixin production in the leaves of *B. orellana* (Faria et al., 2020). However, to date, no transcriptomic studies have investigated these carotenoid-related genes on *B. orellana* immature seeds, where bixin biosynthesis initiates.

Bixin is accumulated mainly in the arile of the seeds, where the chromoplasts form osmiophilic bodies, which are then stored in the vacuoles (Louro and Santiago, 2016). The pigments accumulate to occupy the entire volume of the cell (Rodríguez-Ávila et al., 2011b). These specialized cells are ontogenetically related to anastomosed articulated laticifers that produce reddish latex rich in carotenoids (Almeida et al., 2021). Only 30% of the dry weight in the aril is composed of bixin; the remaining 70% comprises primarily lipids, carbohydrates, and proteins (Carvalho et al., 2010). Latex composition has been described in plant medicinal species and *Hevea brasiliensis*, which is the natural source of rubber. These complex fluids consist of specialized metabolites, like terpenes, alkaloids, or phenolics (Gracz-Bernaciak et al., 2021). According to the above mentioned, bixin biosynthesis might be a complex phenomenon that involves not only the enzymatic activation of certain genes but also the compartmentalization and biogenesis of specialized cells and the production of high amounts of latex. Therefore, this study addresses the transcriptomic analysis of development seed stages in phenotypically contrasting accessions of *B. orellana*. Enrichment and network analysis were employed to explore the main pathways and identify the genes involved in the differential production of bixin and other associated compounds in the specialized gland cells during seed development.

## 2 Materials and methods

### 2.1 Sample collection and preparation

**Figure 1:**
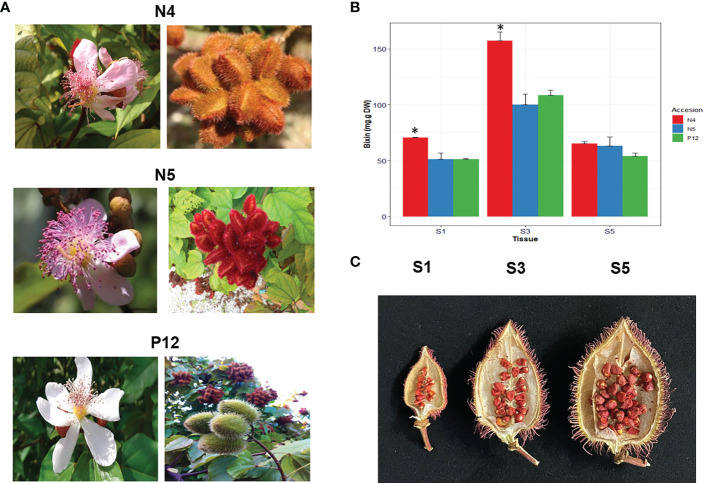
*B*. *orellana* morphotypes and bixin accumulation. **(A)** Flowers and fruits show the characteristics of the accessions used in the current study. **(B)** Bixin accumulation in the different seed development by stage in the different *B*. *orellana* accessions show significant differences (*) in the N4 accession. **(C)** Representative S1, S3, and S5 seed stage in the N4 accession.

Representing *B. orellana* accessions were selected from groups A, B and C based on molecular SNPs characterization by (Trujillo-Hdz et al., 2016). *B. orellana* plants selected were “P12” from A, “N4” from B and “N5” from C. These three accessions were chosen since are representative of the three morphotype groups identified by the SNPs analysis in 32 different accessions. Seed stage selection was set up according to two previous studies. The first one, the study of Cárdenas-Conejo et al. (2015) development in five seed developmental stages where it was observed that the level expression of carotenoid genes and apocarotenoid genes (CCDs, involved in the start of bixin synthesis) was significant, increasing in the stages S3-S4 and dramatic down in the stage S5. In the second one, Carballo-Uicab et al. (2019) analyzed five developmental seed stages in two contrasting *B. orellana* accessions. This analysis shows a high carotene-related gene correlation between seed development and bixin accumulation. Therefore, S1, S3, and S5 stages were chosen to conduct the transcriptomic analysis. Samples of young leaves (recently developed) and three stages of seed development were harvested in *B. orellana* plants cultivated at a commercial plantation in Chicxulub, Yucatán, Mexico (**Figure 1A**). S1 corresponded to those between 0 and 7 Days Post Anthesis (DPA); S3 to those from 14 to 21, and S5 to those with more than 28 DPA, respectively (**Figure 1C**) (Carballo-Uicab et al., 2019). The collected fresh tissues were immediately frozen in liquid nitrogen and stored at -80 °C until analysis. Total RNA was isolated as described by (Rodríguez-Ávila et al., 2009).

### 2.2 Bixin quantification in seeds

The bixin contents was analyzed in seeds throughout their developmental process(Carballo-Uicab et al., 2019). Extracts from 10 mg of frozen, dried, and powdered seed tissue were mixed with 0.8 mL of water/methanol 1:1 (v/v). The mixture was shaken for one h at room temperature and mixed with 800 µL of chloroform. The tubes were vortexed and centrifuged at 9,615 g for 10 min at 4 °C (Sorvall Leyend Micro 21R centrifuge; Thermo Scientific, Waltham, MA, USA), and the bottom phase (nonpolar extract) was filtered using Millex-GV13 filters (Durapore PVDP, 13 mm diameter, 0.22 µm pore size; Millipore, Billerica, MA, USA). The extract was dried under nitrogen gas and stored at -80 °C until analysis.

The analysis of bixin was carried out using HPLC-PDA (photodiode array). The sample of each dried extract was dissolved with 500 µL of dimethyl sulfoxide (DMSO), this was taken 100 µL and put in another tube with 400 µL of DMSO (the dilution was 5x) was shaken and finally, 5 µL was injected into a Hypersil ODS C-18 reversed phase column (25 cm x 4.6 mm; 5 mm diameter beads. The mobile phase consisted of water with 0.1% formic acid, pH 3 (solvent A), and acetonitrile (solvent B). The column was developed as follows: Step 1, 95% solvent A at injection for 5 min; Step 2, linear increase to 95% solvent B in 25 min; Step 3, 95% solvent A for 4 min; and Step 4, return to 100% solvent B. The bixin in the samples was detected at 450 nm on an Agilent 1200 at a Flow rate of 0.4 mL/min at 20 °C. A bixin standard (ChromaDex, Irvine, CA, USA) was used to generate a calibration curve. The bixin was quantified based on their column retention time relative to the known bixin standard and by the absorption spectra of individual peaks.

### 2.3 Illumina sequencing and processing Illumina data

Total RNAs from the different tissues of young leaves and three stages of seed development (S1, S3 and S5) were used for the construction of 36 indexed cDNA libraries (3 plants; 4 tissues; 3 biological replicates). KAPA Stranded mRNA-Seq Kit Illumina platform was used for libraries preparation (KAPA Biosystems: KR0960). The quantity and quality of cDNA libraries were checked on an Agilent 2100 Bioanalyzer DNA chip (Agilent Technologies Inc., Santa Clara, CA). Libraries were single-end sequenced with 50 cycles in three lanes of the Illumina HiSeq 3000 platform (~350 million reads total per lane). Thirty million reads are calculated per library. The preparation of raw Illumina reads was carried out with a single script called Allprep.py designed by Meric Lieberman from Comai laboratory (Liberman, 2016), all the reads with “N” and quality cut off < 20 were deleted.

### 2.4 Reads alignment to reference transcriptome and differential gene expression analysis

Reads were aligned to reference the transcriptome published by (Cárdenas-Conejo et al., 2015). The alignment to reference achiote transcriptome was carried out with a single python script called bwa-doall1.py designed by Comai laboratory (http://comailab.genomecenter.ucdavis.edu/index.php/Bwa-doall), this python script is based on Burrows-Wheeler Aligner 2 software package (BWA 2) (Li and Durbin, 2009) and SAM tools (Li et al., 2009). The count table was extracted from SAM files using the python scripts merge_tables.py (Wheeler, 2019) and countxpression.py (De Wit et al., 2012). Expression levels were calculated using TPM (Transcripts per million). Differential Gene Expression analysis was carried out with the DESeq2 package (Love et al., 2014); genes were considered differentially expressed if |Log2 Fold change| ≥ 1 and Padj ≤ 0.05. Pearson correlation analysis was performed among bixin content and gene expression levels (TPM) by using the corrplot package and reordering the correlation matrix with hclust method (average) in rstudio.

### 2.5 Gene enrichment analysis

All diferentially expressed genes (DEGs) were divided into three comparisons (P12 vs. N5, P12 vs. N4, and N4 vs. N5). The first comparison component regulated all up-or down-regulated genes in the following description. Functional enrichment and classification of DEGs were performed according to the Kyoto Encyclopedia of Genes and Genomes database (KEGG, http://www.kegg.jp/). KOBAS 2.0 software was used to estimate the statistical enrichment of DEGs in KEGG pathways. The corrected p-value of 0.05 was set as the threshold for the significant enrichment of KEGG pathways. Rich factor (RF) values were considered to analyze the results. Transcription factor prediction analysis was performed by iTAK software (iTAK 1.2). The BLASTX search was carried out between the DEGs and the TAIR 10 database (https://www.arabidopsis.org/). The heat maps in this study were drawn by R software (R-2.15.3-win), and the normalization method was performed using a scale package.

### 2.6 WGCNA and identification of significant modules

Weighted gene co-expression network analysis (WGCNA) was constructed with DEGs (p < 0.05) by R package WGCNA (v1.70) (Langfelder and Horvath, 2008). Gene expression matrices were converted to adjacency matrices with the appropriate soft-thresholding powers. Then, topology overlap matrices were calculated by adjacency matrices. Similar clusters were merged using the blockwiseModules function to divide modules with the expression power=8 and minModuleSize = 50.

Phenotypic traits were categorized in red intensities by the numbers2colors function. Hierarchical clustering and dynamic tree-cut method were used to identify gene clusters. Module eigengene was defined as the first principal component of a module gene expression matrix (Langfelder et al., 2008). The correlation strength between module eigengene and phenotypes identified the modules of interest, including plant accessions, seed development stages, and bixin accumulation. We selected the largest and bixin correlated module for further analysis.

### 2.7 RT-qPCR quantification

Expression analysis was conducted by Real-Time Quantitative Reverse Transcription PCR (Real-Time qRT-PCR). The reaction mixture contained 100 ng of cDNA and the SYBR Green qPCR SuperMix-UDG (Cat. No. 11733046; Invitrogen, Carlsbad, CA, USA). PCR was performed using an iCycler IQ real-time PCR detection system (Bio-Rad, Hercules, CA, USA). Specific primers for each selected gene are provided in **Table S1**. The amplification program included 35 cycles (30 s each) at 95 °C for DNA denaturation, followed by 62.3° or 57.6 °C for primer annealing and 72 °C for the extension. The PCR program included an initial 2 and 4 min periods at 50° and 95 °C, respectively, to activate the polymerase and a final 10 min extension at 72 °C. Alternative primer alignment temperatures corresponded to either target sequence. The expression of the 18S rRNA gene was followed in each sample as an internal reference, taking leaves to standardize comparisons among gene expression of seed development stages. The specificity of the PCR was assessed by the presence of a single peak in the dissociation curve performed after the amplification. Each quantitative PCR experiment was run three times separately and included three replicates in calculating the standard error for each sample. The results were analyzed by the 2-DDCT method (Livak and Schmittgen, 2001) with appropriate validation experiments.

## 3 Results

### 3.1 Bixin accumulation

We selected the three *B. orellana* accessions representing the haplotype groups A, B and C previously characterized by (Trujillo-Hdz et al., 2016). These accessions show differences in the color and the morphology of their fruits and flowers and the bixin content, mainly in the S3 stage of their seeds (**Figure 1**). Significant differences (α=0.05) were found in S3, where the N4 accession showed the highest bixin amounts with 154 mg/g D.W. Bixin amount was similar in S5 in all accessions with about 50 mg/g D.W. (**Figure 1B**).

### 3.2 Transcriptome sequencing

To identify the responsible genes for variation in bixin production and morphological differences among the *B. orellana* accessions P12, N4, and N5, we designed an RNAseq experiment to discover differentially expressed genes. For transcriptome sequencing, we isolated total RNA from young leaf (L), seed development stage 1 (S1), seed development stage 3 (S3) and seed development stage 5 (S5) and constructed a total of 36 index cDNA libraries, three replicates per sample (**Table S2**). After Illumina sequencing and a quality filter, 1,044,700,367 high-quality reads were obtained, representing an average of 29 million raw reads per library (**Table S2**). The raw sequencing data have been submitted to the Short Reads Archive (SRA) under the BioSample accessions SAMN31488112 to SAMN31488147. We aligned high-quality reads to the reference *B. orellana* transcriptome sequenced by (Cárdenas-Conejo et al., 2015); 848,335,204 reads were aligned with approximately 23 million reads per library.

### 3.3 KEEG enrichment analysis reveals pathway involved in cuticular wax biosynthesis

We identified differentially expressed genes at stage S3, where the highest bixin accumulation differences were observed (**Figure 1B**). In general, MEP, carotenoid, and bixin pathways slight expression differences when accession N4 was compared, either to P12 or to N5. This suggested that other pathways could contribute to the contrasting bixin accumulation phenotype.

**Figure 2:**
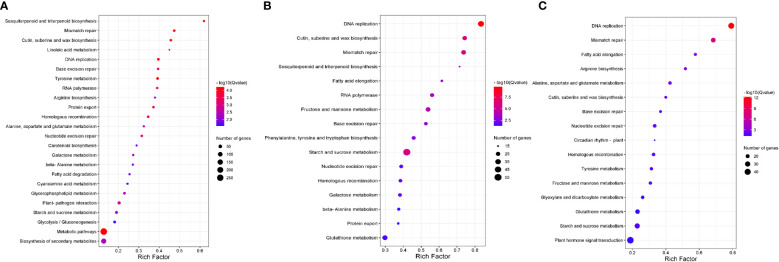
The Kyoto Encyclopedia of Genes and Genomes (KEGG) pathway enrichment analysis of unigenes with higher expression in the S3 stage. The significantly enriched pathways with corrected p-value (q value) < 0.05 are shown. The number indicates the size of the dot, describing the number of unigenes enriched in the pathway. The color bar represents the RF value and indicates the significance of the enrichment. **(A)** KEGG enrichment pathways of the DEGs were exclusively detected in P12 *vs*. N5. **(B)** KEGG enrichment pathways of the DEGs exclusively detected in N5 *vs*. N4. **(C)** KEGG enrichment pathways of the DEGs were exclusively detected in P12 *vs*. N4.

To identify additional pathways involved in bixin accumulation, we performed a Kyoto Encyclopedia of Genes and Genomes (KEEG) enrichment analysis at S3. At a p-value threshold ≤ 0.05, several pathways were highlighted in the three comparisons (**Figure 2**). Of these, three shared pathways caught our attention:

1. “Cutin suberine and wax biosynthesis” was highly enriched in all comparisons (RF≥ 0.4) (**Figure 2**). Here, we found differentially expressed genes encoding enzymes for biosynthesis of monomers of cutin (9,10,18-Trihydroxystereate; fatty acid omega-hydroxylase [EC:1.14.14.80] and peroxygenase [EC:1.11.2.3]), suberin (polyhydroxy α,ɷ-dicarboxylic acid; fatty acid omega-hydroxylase [EC:1.14.14.80]), and wax (aldehyde decarbonylase [EC:4.1.99.5] and alcohol-forming fatty acyl-CoA reductase [EC:1.2.1.84]). Genes for peroxygenase and aldehyde decarbonylase were up-regulated when accession N4 was compared with either P12 or N5.

2. “Fatty acid elongation”, the pathway was highly enriched in the N4 with P12 and N5 comparisons. The gene encoding 3-ketoacyl-CoA synthase [EC:2.3.1.199] was significantly up regulated. This enzyme is involved in cuticular wax and suberin biosynthesis since it catalyzes the condensation of C2 units to acyl-CoA, the first step of fatty acid elongation.

3. “Sesquiterpenoid and triterpenoid biosynthesis” were enriched in the N5 comparisons. In this pathway, we identified the gene encoding Squalene epoxidase as up-regulated in N4. This enzyme is crucial for the regulation of sterols and triterpenoids. Additionally, we identified DEG of enzymes involved in the synthesis of specific triterpenoid compounds such as Germacrene D ((-)-germacrene D synthase), α-Copaene (α-copaene synthase), β-Caryophyllene (β-caryophyllene synthase) and Lupeol (lupeol synthase).

Last, “Mismatch repair” and “DNA replication” were highly enriched in the three comparisons, but we did not analyze them since these pathways are inherent to DNA replication and maintaining genome integrity during seed development.

### 3.4 WGCNA, identification of significant modules and phenotype correlation

**Figure 3:**
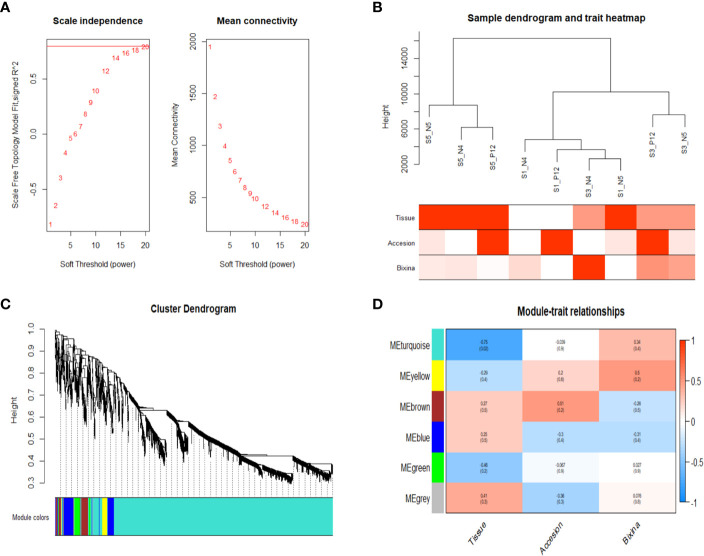
WGCNA showed genes highly related to bixin content. **(A)** Scale independence and average connectivity. **(B)** Clustering dendrogram of 9 samples. Phenotypic traits were categorized in red intensities by the numbers2colors function showing a high bixin accumulation in S3 of the N4 accession. **(C)** Dendrogram showing the turquoise module as the largest. Each branch represents a gene, and each color represents a co-expression module. **(D)** Heatmap of the correlation between module eigengenes and phenotypic characteristics showing the yellow and turquoise modules as the highest correlated with the bixin contents.

WGCNA was used since allows the combining of gene expression with phenotypic data. This analysis was conducted on 3,081 genes obtained after data preprocessing. When the scale-free topology model fit reached 0.8 (R2 = 0.8), a soft thresholding power was 8 (β = 18) (**Figure 3A**). All samples in the network cluster were within the cut-off threshold (height < 1200) with three phenotypic variables used (accession, seed development stage and bixin content) being the S3 of the accession N4 that exhibits a higher bixin content relation (**Figure 3B**). Six co-expression modules were obtained after merging modules with a similarity greater than 0.4 (**Figure 3C**). The module containing the most genes was the turquoise module (2592 genes), followed by the blue module (198 genes), brown module (125 genes), yellow module (74 genes), green module (72 genes) and gray module (14 genes) (**Supplementary Table S3**). The correlation between the co-expression module and the phenotype was analyzed. The results showed that two modules were associated with the bixin phenotype; turquoise (r = 0.74) and yellow (r = 0.75) (**Figure 3D**). However, the turquoise module covers most of the genes exhibiting higher connectivity among them. **Table 1** shows the 25 genes with the highest bixin correlation resulting from the WGCNA analysis. These genes cover isoprenoid, triterpene, carotene, and carotenoid pathways supporting the previous enrichment analysis.

**Table 1:** First 25 highly co-related genes of the bixin related (turquoise) module resulting of the co-expression network analysis.

ID	Annotation (KEEG)	moduleColor	Bixin trait correlation
K09833	PREDICTED: homogentisate geranylgeranyltransferase-like [Citrus sinensis]	turquoise	0.743
K18108	Terpene_synth domain-containing protein[Cephalotus follicularis]	turquoise	0.738
K00344	oxidoreductase family protein [Populus trichocarpa]	turquoise	0.733
K00514	zeta-carotene desaturase [Bixa orellana]	turquoise	0.720
K09840	carotene cleavage dioxygenase 4 copy 2/3/4/5/NCED5 [Bixa orellana]	turquoise	0.698
K03526	4-hydroxy-3-methylbut-2-en-1-yl diphosphate synthase [Bixa orellana]	turquoise	0.697
K00099	1-deoxy-D-xylulose-5-phosphate reductoisomerase [Bixa orellana]	turquoise	0.696
K01770	2-C-methyl-D-erythritol 2,4-cyclodiphosphate synthase [Bixa orellana]	turquoise	0.689
K00797	Spermidine synthase 3 isoform 3 [Theobroma cacao]	turquoise	0.685
K02291	phytoene synthase 1 /2 [Bixa orellana]	turquoise	0.681
K02493	NA	turquoise	0.680
K14157	alpha-aminoadipic semialdehyde synthase-like isoform X1 [Durio zibethinus]	turquoise	0.677
K16221	TCP family transcription factor [Theobroma cacao]	turquoise	0.676
K01662	1-deoxy-D-xylulose-5-phosphate synthase 1/2a/3 [Bixa orellana]	turquoise	0.670
K14182	terpene synthase 12 [Tripterygium wilfordii]	turquoise	0.666
K08869	Kinase superfamily protein isoform 2 [Theobroma cacao]	turquoise	0.661
K03527	4-hydroxy-3-methylbut-2-enyl diphosphate reductase [Bixa orellana]	turquoise	0.656
K13237	Short-chain dehydrogenase-reductase B [Theobroma cacao]	turquoise	0.655
K03255	PREDICTED: protein TSS [Theobroma cacao]	turquoise	0.654
K10841	protein CHROMATIN REMODELING 8 [Manihot esculenta]	turquoise	0.654
K06901	AZA-guanine resistant1 [Theobroma cacao]	turquoise	0.652
K09013	ABC transporter I family member 6, chloroplastic [Herrania umbratica]	turquoise	0.651
K00128	aldehyde dehydrogenase 3H1/2B7/3l1/2B4/3F1/3F2 [Bixa orellana]	turquoise	0.646
K13789	geranylgeranyl diphosphate synthase [Bixa orellana]	turquoise	0.645
K00487	Cytochrome P450 [Corchorus olitorius]	turquoise	0.643

### 3.5 MVA and MEP gene pathway analysis

**Figure 4:**
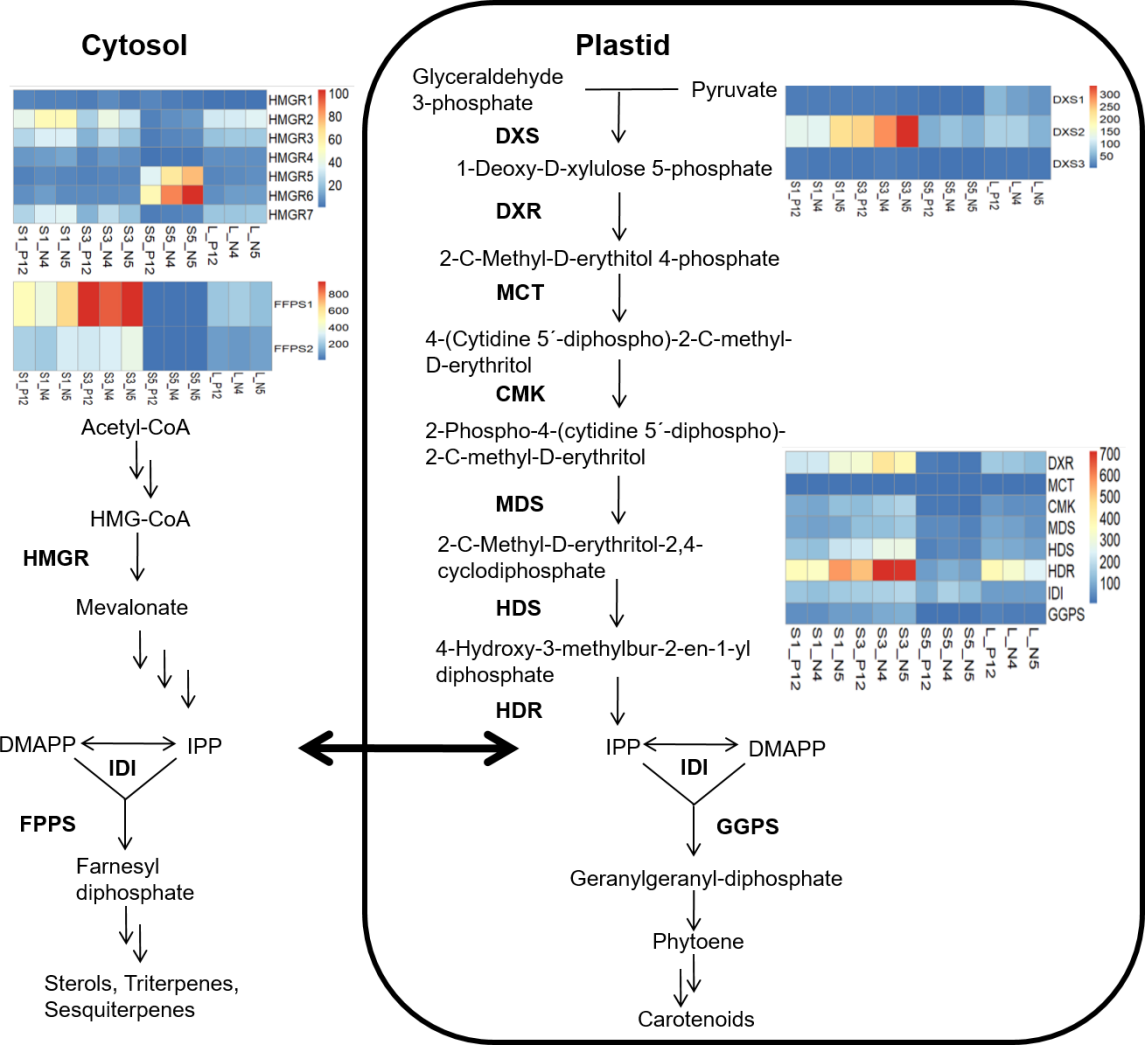
MAV and MEP pathways supply the biosynthesis of terpene and carotene molecules during seed development in different *B*. *orellana* accessions. Mature leaves of each accession were also analyzed to show the gene activation in the biosynthesis of photosynthetic molecules. Genes with well-known functions of regulatory checkpoints in the MAV and MEP pathways are showed. Heat maps show the TPM values of each gene. *Bo* nomenclature from gene names was removed to simplify the images.

To better understand the terpene/sesquiterpene biosynthetic process, a metabolic pathway enriched in all accession comparisons, we examined the key genes of the mevalonate (MVA) and the 2C-methyl-D-erythritol-4-phosphate (MEP) pathways. These pathways provide a wide diversity of isoprenoid molecules in plants (Vranová et al., 2013). Expression levels were determined in all (S1, S3, and S5) stages of seed development and the leaves of each accession using the TPM normalized metric (**Table S4**). We selected genes encoding the enzyme 3-hydroxy 3-methylglutaryl Coenzyme A reductase (HMGR), which produces mevalonate and is involved in the early triterpene pathway (Narváez et al., 2001), and the gene encoding farnesyl diphosphate synthase (FFP), which is involved in the first stage of sesquiterpenes (C_15_) biosynthesis (Durairaj et al., 2019). Seven different *HMGR* orthologues were identified. *BoHMGR5* and *BoHMGR6* were highly activated in all accessions’ final phase (S5) of seed development. In addition, we detected two paralogous genes encoding *BoFPS*. *BoFPS1* was highly upregulated in S3 during the maximum seed development in all accessions (**Figure 4**).

In the MEP pathway, we examined the gene encoding 1-deoxy-D-xylulose 5-phosphate synthase (DXS) enzyme, the first enzyme involved with the synthesis of 1-deoxy-D-xylulose 5-phosphate (DXP) from pyruvate and D-glyceraldehyde 3-phosphate. We identified three paralogs of this gene, although only *BoDXS2* was upregulated, mainly in S3. Other eight genes encoding enzymes of the MEP pathway were also analyzed; 1-deoxy-D-xylulose 5-phosphate reductoisomerase (HDR), 4-diphosphocytidyl-2-C-methyl-D-erythritol synthase (MCT), 4-diphosphocytidyl-2-C-methyl-D-erythritol kinase (CMK), 2-C-methyl-D-erythritol 2,4-cyclodiphosphate synthase (MDS), 4-hydroxy-3-methylbut-2-enyl diphosphate synthase (HDS), 4-hydroxy-3-methylbut-2-enyl diphosphate reductase (HDR), isopentenyl diphosphate isomerase (IDI) and geranylgeranyl diphosphate synthase (GGPS) (Tarkowská and Strnad, 2018; Zhou and Pichersky, 2020). Only the *BoDXR* and *BoHDR* genes showed a similar tendency between them, being the *BoHDR* the more up-regulated, mainly in the end stage of the seed development (S5) and in the best pigment producer accessions N4 (**Figure 4**). The accumulation of plastidial isoprene molecules might be related to the accumulation of pigment carotenoids (bixin) in the last seed stages. Therefore, a further carotene analysis was conducted.

### 3.6 Correlation analysis

**Figure 5:**
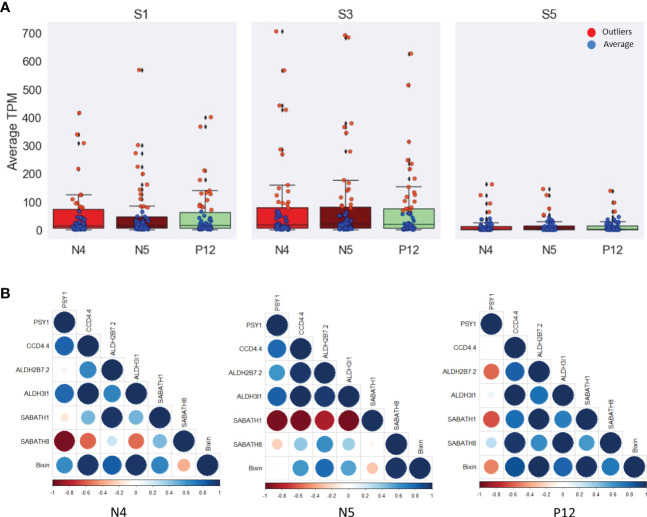
TPM analysis of the 57 genes related to isoprene, terpene, carotene, and carotenoid pathways. **(A)** Box plots showing the average (blue) and outlier (red) genes during the seed development. S3 exhibits most of the outlier genes. **(B)** Heat maps showing the spearman correlation among the more highly correlated genes with the bixin in each *B*. *orellana* accession. The low correlated *PSY1* gene was selected to show the differences in the correlation values. The head arrow indicates the bixin. *Bo* nomenclature from gene names was removed to simplify the images.

WGCNA allows identifying genes associated with bixin contents belonging to the isoprene (MVA and MEP), terpene, carotene, and carotenoid pathways. The sixth gene identified as belonging to these pathways were included. Special care was taken to include to the carotene-related genes encoding phytoene synthase (*BoPSY*), phytoene desaturase (*BoPDS*), ζ-carotene desaturase (*BoZDS*), lycopene β-cyclase (*BoβLCY*), and lycopene ϵ-cyclase (*LϵCY*). In addition, apocarotenoid (*BoCCDs*, *BoALDHs*, and *BoMETs*) related genes were also included since high amounts (80%) of C_25_ apocarotenoid bixin occurs in the seeds (Rivera-Madrid et al., 2006). Most of these genes have a similar range of TPM (< 100) in the initial and intermediate seed development stages (S1 and S3; **Figure 5A**). Contrary to expectation and regardless of the accession analyzed (**Table S4**), these carotene-related genes displayed relatively low expression at the S5 stage. Some genes exhibited outlier values in all stages and accessions, mainly in the S3 (**Figure 5A**). These genes are *BoDXS2, BoDXR*, and *BoZDS*, with the highest values (>300 TPM). The CCD genes (*BoCCD1-3*, *BoCCD1-2*, *BoCCD4-3*) with >50 TPM, the ALDH genes (*BoALDH3H1-1*, *BoALDH10A8*, *BoALDH6B2-1* and *BoALDH7B4*) with >100 TPM, and some METs (*BoSABATH3* and *BoSABATH4*) with >10 TPM (**Table S4**).

Correlation analysis was conducted with the 61 genes selected. **Supplementary Table S5** shows all correlations among these genes and the bixin content by seed stage. A selection of the highest correlated genes of these pathways is given in **Figure 5B**. *CCD4.4*, *ALDH2B7.2*, *ALDH3I1*, *SABATH1* and *SABATH8* were the genes that exhibited the highest bixin content correlation in the different accessions, particularly with the N4 accession. Specifically, this accession shows the highest bixin correlation (> 0.9) with the *BoPSY2*, *BoPDS1*, and *BoZDS* genes. On the contrary, *BoLϵCY* displayed the lowest correlation to bixin concentration. A similar tendency was observed in the N5 accession. Although gene correlations were moderate (> 0.8), in the P12 accession, *BoPSY2*, *BoPDS1*, and *BoZDS* genes displayed a good correlation to bixin, while the *BoPSY1* and *BoLϵCY* genes displayed negative correlation. Thus, *BoPSY1* was selected as representative of the genes with a low correlation to bixin in **Figure 5B**. The lycopene cyclase genes generally showed a reduced or negative bixin correlation during seed development in all *B. orellana* accessions (**Table S5**). Investigating apocarotenoid related genes, we analyzed the CCD genes. In the N4 accession, most of the CCD genes positively correlated to bixin, particularly *BoCCD4-1* (0.8), while *BoCCD4-5* and *BoCCD1-4* did not. In the N5 accession, all the CCD gene members exhibited high bixin correlation (> 0.7), particularly *BoCCD4-1, BoCCD4-2*, and *BoCCD4-4* (0.9). In the P12 accession, only *BoCCD4-1* and *BoCCD4-4* displayed a high correlation (0.8) (**Figure 5B**). The N4 accession, which displays the highest bixin accumulation in the S3 stage, showed only a moderate correlation of bixin with most of the *BoCCD* genes. This suggested that bixin production might be regulated downstream in the apocarotenoid pathway. Therefore, other apocarotenoid-related genes, such as *BoALDHs* and *BoMETs* (*BoSABATH*) were also evaluated. Few *BoALDH* gene members correlated highly with bixin in the different accessions. In N4, only *BoALDH2C4* displayed good correlation (0.8), whereas in N5 and in P12 only the *BoALDH2B4*, *BoALDH3I1* and *BoALDH3H1-1*, and the *BoALDH3I1*, *BoALDH2B4* and *BoALDH2C4* gene members, respectively, displayed high bixin correlation (> 0.8). For the three *B. orellana* accessions analyzed, most of the *BoALDH* gene members displayed reduced bixin correlation during seed development. Regarding *BoMET* gene members, only the N4 accession exhibited a high bixin correlation with several *BoMET* genes, including *BoSABATH6*, *BoSABATH4*, *BoSABATH3* and *BoSABATH5* (0.9). In the P12 accession, only *BoSABATH5* exhibited high bixin correlation (0.8) whereas than in N5 only *BoSABATH3* showed a moderate bixin correlation (0.5) **Table S5**). These results indicate that both the *BoALDH* and *BoMET* genes contributed to bixin accumulation during seed development.

Considering these results with the different carotene- and apocarotenoid-related genes (*BoCCDs*, *BoALDH* and *BoMETs*) it is possible that specific carotene-related genes, mainly *BoPSY2*, *BoPDS1* and *BoZDS*, some *BoCCD* genes as *BoCCD1-4*, *BoCCD4-1*, *BoCCD4-3* and *BoCCD4-4*, and some *BoALDH* genes as *BoALDH3I1*, *BoALDH2B4* and *BoALDH3H1-1*, and specific *BoMET* genes as *BoSABATH3* and *BoSABATH5*, might participate in bixin biosynthesis during seed development in these *B. orellana* accessions (**Figures 4**, **5** **;** **Table S5**).

**Figure 6:**
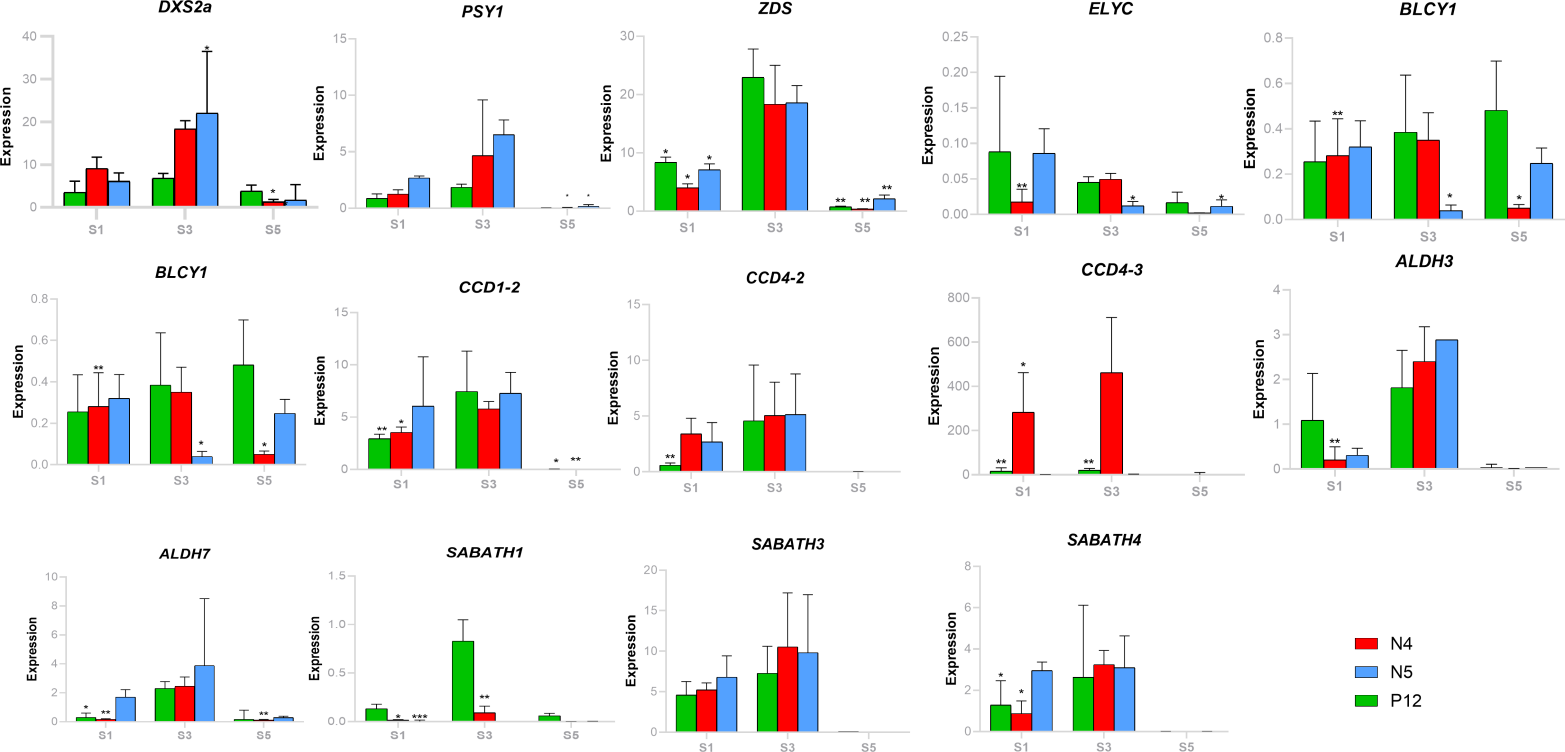
Some carotenoid-related genes’ expression patterns (RT-PCR) during seed development in the different *B*. *orellana* accessions. *Bo* nomenclature from gene names was removed to simplify the images. Asterisk indicates significant Tukey´s groups.

Finally, RT-qPCR analysis was conducted to support the TPM expression analysis with selected genes (**Figure 6**). *BoDXS2* expression exhibited upregulation in the S3 for all accessions confirming the RNAseq data. Similarly, *BoZDS* and *BoPSY* exhibited a strong upregulation in S3, supporting their role in the production of substrates for carotenoid biosynthesis. Cyclase genes were more upregulated in the initial seed development stages (S1 and S3), mainly *BoLβCY2*, supporting the previous bixin correlation analysis and modifying the idea that only the C_40_ linear substrates occur in the S5 development stages. More research will be necessary to clarify the role of the cyclic substrates in the secretions of reddish latex and bixin of the seeds of *B. orellana*. Among the apocarotenoid-related genes, *BoCCD1-2* of the subfamily one was highly regulated in the S1 and S3 in all accessions. *BoCCD4-2* and *BoCCD4-3* of the subfamily four were mainly upregulated in the S3 phase in high correlation with bixin accumulation. *BoALDH* and some *BoMETs* genes also were up-regulated in the mature seed stage (S3), most notably in all accessions *BoALDH3* and *BoSABATH4*. The activation of diverse *BoCCDs*, *BoALDH*, and *BoMETs* genes in the S3 stage suggested a cooperative contribution to the carotenoid accumulation of *B. orellana*.

## 4 Discussion

### 4.1 KEEG Enrichment analysis reveals genes involved with the biogenesis of specialized cells and the production of latex

The high enrichment observed in the genes related to the metabolic pathways of “Cutin suberin and wax biosynthesis”, “Fatty acid elongation,” and “Sesquiterpenoid and triterpenoid biosynthesis” in the *B. orellana* accessions comparison analysis suggests an important role of these pathways in the phenotypic differentiation among these accessions. The three accessions showed similar bixin contents in S5, but significant bixin amount differences in S3, particularly for accession N4. The fruit morphology in P12 and N4 showed similar structures during the fruit and seed development (Tamayo-García et al., 2022). Therefore, the bixin differences observed likely resulted from cellular or metabolic changes in the seeds. Triterpene enrichment might supply sterols used in membrane biogenesis (Zhou and Pichersky, 2020). Membrane biogenesis, which includes genes associated with “Fatty acid elongation”, occurs during the seed accumulation and the cell development glands in this tissue (Almeida et al., 2021). These initial gland cells begin a process of coalescence with the adjacent cells where the cell walls among them are degraded, forming plurinucleated cells with frequent spherical vacuoles. These vacuoles fuse during seed development to form a single central vacuole. Abundant chromoplasts are also formed in association with the endoplasmatic reticulum and are remarkably close to the large central vacuole (Almeida et al., 2021). Sesquiterpene enrichment in the seeds also might be associated with the production of herbivore-repellent molecules (Durairaj et al., 2019) or in the biosynthesis of isoprenoid molecules important in the reddish latex in which bixin is incorporated. Diverse isoprenoids, besides bixin, have been identified from *B. orellana* seeds, including the all-E-geranylgeraniol, farnesylacetone, geranylgeranyl octadecanoate, geranylgeranyl formate, and the δ-tocotrienol (Jondiko and Pattenden, 1989; Lancaster and Lawrence, 1995). In fact, in the chromoplast of the cell glands, lipid droplets (Almeida et al., 2021) accumulate and form eletrondense plastoglobuli (Louro and Santiago, 2016). Microscopy analysis indicates that chromoplasts discharge very electrondense lipid material directly into the close central vacuole. The second pathway enriched in the accession comparisons was that limonene biosynthesis. This compound is a monoterpene in flowering plants that is synthesized and stored in specialized anatomical structures such as glandular trichomes, secretory cavities, and root resin ducts (Bergman and Phillips, 2021). Limonene is produced through the MEP pathway (Bergman and Phillips, 2021).

WGCNA supports the results obtained with the enrichment analysis. The turquoise module covers most genes (2,592) and high correlation (r = 0.74) with the bixin phenotype. This module shows genes distributed in different branches and weak correlation, indicating the diversification of regulatory mechanisms for bixin accumulation. Gene diversification implies in the production of specialized metabolites have been documented by using WGCNA during the production of the aromatic compound in *Dendrobium catenatum* (Lige et al., 2022). Particularly, this strategy allows the identification of diverse genes highly related to bixin accumulation. These genes belong to isoprene (MAV and MEP), terpenes, carotenes, and carotenoid pathways. MAV and MEP pathways might contribute to non-carotenoid molecules that accumulate in reddish latex. The exact chemical composition of the reddish secretions produced by *B. orellana* remains to be analyzed. Additional investigations of the non-carotenoid molecules are needed to clarify their role in bixin accumulation in *B. orellana* seeds. In the same way, the enrichment analysis also allows identifying diverse genes related to the other specialized metabolisms involved with the production of latex and the transport of bixin, which must also be considered in further studies.

TPM analysis highlighted the role of the regulatory genes of the MVA pathway, *BoHMGR* (Falginella et al., 2021), and *BoFFP* (Durairaj et al., 2019). The *BoHMGR5* and *BoHMGR5* paralogues were upregulated, peaking in S5 of seed development in all accessions, whereas *BoFFPS1* paralogs were highly active in the S3, a critical stage for seed development. As previously described, terpene biosynthesis, particularly sterols biosynthesis, might be involved in cell differentiation (Falginella et al., 2021). *BoHMGR* transcript activation was previously documented in fruit development, including that of the seeds in *B. orellana* (Narváez et al., 2001), prompting the suggestion of a central role in growth and development. No previous reports on *BoFPS* in *B. orellana* were found in the literature. This enzyme participates in the first stage of sesquiterpenes (C_15_) biosynthesis (Durairaj et al., 2019). In general, sesquiterpenes are produced by sesquiterpene synthases in the cytosol, where FPP is synthesized (Vranová et al., 2013; Zhou and Pichersky, 2020). Importantly, some isoprenoid precursors of carotene or precursors of reddish latex components might be available in the cytosol when the chromoplasts discharge the electrondense lipid material into the lateral or central vacuole in the specialized gland cells (Almeida et al., 2021). More research is necessary to ascertain the role of the *BoFPS* and the sesquiterpene in the specialized cells of the seeds of *B. orellana*. TPM analysis also highlighted MEP regulatory genes. Plastid isoprenoids contribute to carotene (Zhou and Pichersky, 2020); therefore, more key genes were selected in this analysis. Specifically, WGCNA shows that several gene members of the MEP pathways are highly correlated to bixin content. *BoDXS2* paralogs were upregulated, mainly in the S3 of the N5 accession. DXS catalyzes the first step by synthesizing 1-deoxy-D-xylulose 5-phosphate from pyruvate and D-glyceraldehyde 3- phosphate and acts as a major flux-control step of the MEP pathway in plants (de Luna-Valdez et al., 2021). The current study identified three *BoDXS* paralogues; the occurrence and differential expression of different *DXS* paralogues has been previously documented, supporting the notion that each *DXS* class has a specific role in the synthesis of different isoprenoids (de Luna-Valdez et al., 2021). In the N4 and N5 accessions, *BoDXR* was also up-regulated, mainly in the S5 stage. This gene encodes DXR, which catalyzes the conversion of 1-deoxy-D-xylulose 5-phosphate to 2-C-methyl-D-erythritol 4-phosphate, representing the first committed enzymatic step of the MEP pathway (Lee et al., 2022). *BoHDR* transcripts also showed a similar tendency peaking in the S5, mainly in the N4 and N5 accessions. The enzyme encoded by this gene acts at the end of the MEP pathway with the production of IPP and DMAPP from 4-hydroxy-3-methylbut-2-enyl diphosphate, and its regulation has been associated with plastidic isoprenoid biosynthesis (Lee et al., 2022). The activation of the committed key MEP genes (*BoDXS2*, *BoDXR* and *BoHDR*) indicates a strong plastidic regulation to produce plastidic isoprenoids, probably carotenoid pigments, in the immature seeds of *B. orellana*. Therefore, a detailed analysis of most carotene and apocarotenoid related genes was conducted.

### 4.2 Role of the carotene and apocarotenoid related genes

Some of the highest bixin-correlated genes identified in the WGCNA approach were involved in the carotene pathway. The carotene related genes selected include *BoPSY*, which encodes the phytoene synthase enzyme that catalyzes the further condensation of two GGDP molecules to produce the colorless phytoene molecule. Further, we observed activation of the genes encoding phytoene desaturase (PDS) and ζ-carotene desaturase (ZDS). These genes catalyze four phytoene desaturations steps to produce ζ-carotene and lycopene, respectively. Lycopene undergoes two cyclization reactions forming α- and β-carotene. Lycopene β-cyclase (Lβ CY) introduces two β-rings to the ends of the lycopene carbon chain, forming β-carotene, whereas LβCY and lycopene ϵ-cyclase (LϵCY) form α-carotene (Simkin, 2021). In general, these genes show an upregulation in S1 and S3 with a slight expression reduction in S5. *BoPSY2*, *BoZDS*, and *BoLβCY* and *Bo-LϵCY* genes were upregulated. Particularly, *BoZDS* values were the highest. This is a key enzyme involved in the lycopene biosynthesis (Simkin, 2021) and the high values obtained indicate high lycopene accumulation overall in S1 and S3, concurrent with apocarotenoid biosynthesis. In the current study, expression of *BoPSY2*, *BoPDS1*, and *BoZDS* genes was highly correlated with bixin accumulation, regardless of the *B. orellana* accession. These genes are closely connected to lycopene production in plastids (Simkin, 2021). Lycopene is also considered the precursor of bixin through the selective cleavage of BoCCDs enzymes (Bouvier et al., 2003; Carballo-Uicab et al., 2019; Us-Camas et al., 2022) to form bixin aldehyde, and then this molecule is oxidized by an *BoALDH* to produce norbixin, and finally, the norbixin product is methylated by *METs* to produce bixin (Jako et al., 2002; Bouvier et al., 2003). Only *BoCCDs* involved with the bixin production were included in this analysis; those belonging to the group of the 9-cis epoxycarotenoid dioxygenases involved with the biosynthesis of ABA were discarded. The CCD enzymes are generally endowed with different cleavage specificities and act on different substrates (Ahrazem et al., 2016). In the current study, *BoCCD1-4* and several *BoCCDs* of the subfamily 4 (*BoCCD4-2*, *BoCCD4-3*, and *BoCCD4-4*) show a high correlation to bixin, particularly in S3 and S5, suggesting an active role in the apocarotenoid biosynthesis. In general, diverse *BoCCD* gene members of both subfamilies have demonstrated involvement with the bixin biosynthesis (Cárdenas-Conejo et al., 2015; Carballo-Uicab et al., 2019; Us-Camas et al., 2022), and its subcellular compartmentalization has been hypothesized (Cárdenas-Conejo et al., 2015). *BoCCD4-3* gene expression has also been correlated with the increase in bixin in the leaves of another accession (Faria et al., 2020). Moreover, it has also been demonstrated that *BoCCD4-3* can also cleave cyclic carotenes to produce C_20_ apocarotenoid, although not bixin in heterologous system (Frusciante et al., 2021). In addition, some of the *BoCCDs* genes of the subfamily 1, mainly *BoCCD1-4*, also exhibit a high bixin correlation during seed development in all accessions of this study. The occurrence *in vivo* of different up-regulated *CCDs* members and their ability to cleave lycopene and cyclic carotenes to produce apocarotenoids may be explained by spatio-temporal participation in bixin biosynthesis (Us-Camas et al., 2022) or by a *BoCCDs* its differential distribution in the plastid or the cytosol (Cárdenas-Conejo et al., 2015), probably during reddish latex production and storage from chromoplasts to central vacuole in the specialized gland cells (Almeida et al., 2021). More research must be done to clarify this important key step in bixin biosynthesis.

Different *BoALDH* paralogues, *BoALDH3I1*, *BoALDH2B4* and *BoALDH2C4*, are correlated with the bixin accumulation suggesting an important role in apocarotenoid biosynthesis. The relationship between increased of the *BoALDH3I1* expression with the increased of the bixin content has been previously reported (Cárdenas-Conejo et al., 2015; Faria et al., 2020). Although some *BoALDH* paralogues were also up-regulated, they were not correlated with bixin. Relevant to this observation, *BoALDH* are very diverse and can be placed in 24 families, which may occur in mitochondria or the cytosol and have physiological importance in plant development and environmental stress adaptability(Islam and Ghosh, 2022).

The *BoMETs* genes, *BoSABATH5* and *BoSABATH3*, exhibited high bixin correlation in P12 and N5, respectively. Only the N4 accession exhibited high bixin correlation with several *BoMET* genes, including *BoSABATH6*, *BoSABATH4*, *BoSABATH3* and *BoSABATH5*. High expression in mature seeds has been reported for the *BoSABATH4* gene in the accession “Peruana roja” of *B. orellana* (Cárdenas-Conejo et al., 2015). Diverse *BoSABATH* members were identified. These encode diverse enzymes that catalyze the methylation of carboxylic acids and nitrogen atoms, have strict substrate specificities and are involved in a wide diversity of functions in the plants (Wei et al., 2021). A more detailed analysis of bixin biosynthesis, specifically at the cell-specific level, would be very important. In particular, biochemical, histological, *in situ* hybridization, and heterologous strategies must be incorporated in future studies to gain information on the complex profile of metabolites produced in *B. orellana*. In this sense, we also proposed considering those genes related to synthesizing the triterpenes and latex that accompany the bixin in further studies.

In conclusion, the results show a high regulatory complexity during the reddish latex and bixin biosynthesis in the specialized cell glands of the immature seeds of *B. orellana*. The relationship among the biosynthetic pathways of the different molecules (i.e., apocarotenoids, terpenes, and polysaccharides) that occur in these specialized cells, the probable cell compartmentalization of different enzymes during the reddish latex production and storage, and the occurrence of different *BoCCDs*, *BoALDHs* and *BoMETs* gene members with different specificities on different carotene substrates indicate that more *in vivo* studies in the productive *B. orellana* cultivars should be useful to understand the metabolic context for bixin production in this important crop.

## Data availability statement

The original contributions presented in the study are publicly available. This data can be found here: NCBI, PRJNA895001.

## Author contributions

YC-C and JN-Z carried out the bioinformatics analyses. YC-C, JN-Z, RU-C, and PE-T carried out the data analysis. LC cured and assembled the raw reads. MA-E collected and process the vegetal material. VC-U and MA-E conducted the RT-PCR analysis. LC provided genomic expertise and training. RR-M obtained the funds. RR-M conceived, designed, and supervised. All authors contributed to the article and approved the submitted version.
